# Bioactivities of Ketones Terpenes: Antifungal Effect on *F. verticillioides* and Repellents to Control Insect Fungal Vector, *S. zeamais*

**DOI:** 10.3390/microorganisms3040851

**Published:** 2015-11-12

**Authors:** Romina P. Pizzolitto, Jimena M. Herrera, Yesica P. Zaio, Jose S. Dambolena, Maria P. Zunino, Mauro N. Gallucci, Julio A. Zygadlo

**Affiliations:** 1 Instituto Multidisciplinario de Biología Vegetal (IMBiV-CONICET), Universidad Nacional de Córdoba—(UNC), Avenida Vélez Sarsfield 1611, X5016GCA Córdoba, Argentina; E-Mails: rpizzolitto@imbiv.unc.edu.ar (R.P.P.); jimenita_herrera@yahoo.com.ar (J.M.H.); yesicazaio14@gmail.com (Y.P.Z.); pauzun@efn.uncor.edu (M.P.Z.); jzygadlo@efn.uncor.edu (J.A.Z.); 2 Instituto de Ciencia y Tecnología de los Alimentos (ICTA), Facultad de Ciencias Exactas, Físicas y Naturales (FCEFyN), UNC, Avenida Vélez Sarsfield 1611, X5016GCA Córdoba, Argentina; 3 Centro de Investigaciones y Transferencia de Santiago Del Estero (CITSE-INBIONATEC) El Zanjón, ruta 9 Km. 1134, G4200AQF Santiago Del Estero, Argentina; E-Mail: galu2805@hotmail.com

**Keywords:** *F. verticillioides*, *S. zeamais*, α,β-unsaturated ketones, Q-SAR

## Abstract

Maize is one the most important staple foods in the world. However, numerous pests, such as fungal pathogens, e.g., *Fusarium verticillioides*, and insects, such as *Sitophlilus zeamais,* attack maize grains during storage. Many *F. verticillioides* strains produce fumonisins, one of the most important mycotoxin that causes toxic effects on human and animal health. This situation is aggravated by the insect fungal vector, *Sitophlilus zeamais*, which contributes to the dispersal of fungal spores, and through feeding damage, provide entry points for fungal infections. The aim of this study was to evaluate *in vitro* bioassays, the antifungal activity on *F. verticillioides* M3125 and repellent effects against *S. zeamais* of ketone terpenes. In addition, we performed Quantitative structure–activity relationship (Q-SAR) studies between physico-chemical properties of ketone terpenes and the antifungal effect. Thymoquinone was the most active compound against *F. verticillioides* (Minimum Inhibitory Concentration, MIC: 0.87) affecting the lag phase and the growth rate showing a total inhibition of growth at concentration higher than 2 mM (*p* < 0.05). The Q-SAR model revealed that the antifungal activity of ketone compounds is related to the electronic descriptor, Pi energy. Thymoquinone showed a strong repellent effect (−77.8 ± 8.5, *p* < 0.001) against *S. zeamais*. These findings make an important contribution to the search for new compounds to control two stored pests of maize.

## 1. Introduction

Maize is one the most important staple foods in the world with an annual production over 700 million metric tons/year [1]. Argentina is the second largest exporter of maize in the world (27 million tons) [2]. Numerous pests, such as fungal pathogens, e.g., *Fusarium verticillioides*, and insects, such as *Sitophlilus zeamais,* attack maize grains during storage (9.6% of maize grain production is lost) [3], causing substantial damage to cereals, manifested as general spoilage. Many *F.* *verticillioides* strains produce fumonisins, one of the most important mycotoxins that cause toxics effects on human and animal health [4,5,6,7]. Consequently, the occurrence of *F. verticillioides* and fumonisins cause great economic losses due to grain quality deterioration. Mycotoxin formation can occur in pre and post-harvest stages. However, a high production of fumonisin takes place during grain storage, when the temperature and the humidity favor fungal growth and secondary metabolite production [8]. This situation is aggravated by the insect fungal vector, *Sitophlilus zeamais* Motschulky (Coleoptera:Curculionidae), an important pest of the stored maize [9], which contributes to the dispersal of fungal spores and through feeding damage provides entry points for fungal infections, favoring mycotoxin production by *F. verticillioides* [10,11].

Synthetic compounds are used as fumigants to protect stored-products [11,12]. However, the extensive use of synthetic pesticides is associated with problems in human health and other negative effects on the environment [13]. In addition, the use of these synthetic fumigants is related to the development of resistant populations of fungi and insects. Modern strategies of biological control of pests are being investigated as alternatives to synthetic pesticides; monoterpenes found mainly as components of essential oils of plants are an alternative. Previous studies reported bioactivities (antifungal, insecticidal and repellence) of terpenes with different structural groups on stored grain pests, with ketones being the most bioactive [14,15,16,17]. However, little is known about the bioactivity of natural ketone compounds against biotrophic pathogens. The aim of this study was to evaluate the antifungal activity on *F. verticillioides* and repellent effects against insect fungal vector, *S. zeamais* of ketone terpenes using *in vitro* bioassays. In addition, we performed Q-SAR studies between physico-chemical properties of ketone terpenes and the antifungal effect.

## 2. Experimental Section

### 2.1. Chemicals

**Figure 1:**
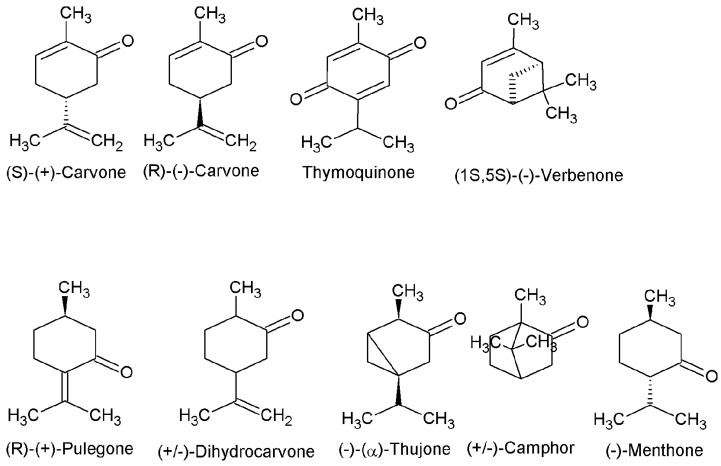
Chemical structures of ketones compounds studied in the present work.

All the ketones compounds used for bioassays were purchased from Sigma Aldrich (Steinheim, Germany). The used ketones compounds are shown in Figure 1: **(*R*)-(+)-Pulegone** (85%) (IUPAC (International Union of Pure and Applied Chemistry) name: (5R)-2-Isopropylidene-5-methylcyclohexanone), **thymoquinone** (99%) (IUPAC name: 2-Isopropyl-5-methyl-1,4-benzoquinone), **(*R*)-(−)-carvone** (98%) (IUPAC name: (5R)-5-Isopropenyl-2-methyl-2-cyclohexen-1-one), **(*S*)-(+)-carvone** (96%) (IUPAC name: (5S)-5-Isopropenyl-2-methyl-2-cyclohexen-1-one, **(−)-menthone** (90%) (IUPAC name: (2S,5R)-2-Isopropyl-5-methylcyclohexanone), **(−)-α-thujone** (>96%) (IUPAC name: (1S,4R,5R)-1-Isopropyl-4-methylbicyclo[3.1.0]hexan-3-one), **(+)-dihydrocarvone** (mixture of “*n*” and “*iso*” isomers) (98%) (IUPAC name: (2R,5R)-5-Isopropenyl-2-methylcyclohexanone), **(1*S*)-(−)-verbenone** (94%) (IUPAC name: (1S,5S)-4,6,6-Trimethylbicyclo[3.1.1]hept-3-en-2-one), **(±) camphor** (96%) (IUPAC name: 1,7,7-Trimethylbicyclo[2.2.1]heptan-2-one). Propionic acid (Sigma-Aldrich Chemical Co.) was used as reference repellent. The (+)-dihydrocarvone and (±) camphor were used as mixture of isomers. The rest of ketone terpenes were used in the diasteropure form.

### 2.2. Antifungal Activity of Ketones

#### 2.2.1. Fungal Culture

The fungal strain *F. verticillioides* M3125, provided by Dr. Robert Proctor (United States Department of Agriculture, Agricultural Research Service, National Center for Agricultural Utilization Research, Peoria, IL, USA), was used for all experiments. It was isolated from maize in California and is a fumonisin-producing strain [18].

#### 2.2.2. Medium and Culture Conditions

The culture conditions were carried out according to Pizzolitto *et al.* [19]. Stock solutions of each ketone were prepared in dimethyl sulfoxide (DMSO), and the appropriate amount to obtain the concentrations of 0.25; 0.5; 0.75; 1.0; 1.5; 2.0 and 3 mM was added to the culture medium. In the control treatments the equivalent amount of DMSO was added to the culture medium.

For the evaluation of antifungal activities, experiments were performed using Czapek–Dox agar in Petri dishes (90 mm). The culture medium was autoclaved at 120 °C for 15 min before cooling at 45 °C an appropriated volume of ketones was added to the media to reach the intended concentrations mentioned above. Inoculum was prepared by growing *F. verticillioides* M 3125 on Czapek agar for 7 days at 25 °C to obtained heavily sporulating cultures. A conidial suspension was placed in aqueous solution, after homogenizing, the suspension was counted using a Neubauer chamber and adjusted to 10^6^ conidia·mL^−1^. Czapek–Dox plates were inoculated centrally with 10 µL of the spore suspension. All the experiments were repeated twice.

#### 2.2.3. Growth Assessment

The antifungal activity was measured as a growth assessment. Two perpendicular diameters of the growing colonies were measured daily (mm) until the colony reached the edge of the plate (approx. 7 days). The radios of the colonies were plotted against time, and a linear regression applied in order to obtain the growth rate as the slope of the line. The effect of each ketone compound on Lag phase for growth, was defined as the time (days) in which each colony reaches 5 mm of diameter [15].

The inhibition percentage was plotted against concentration to each compound. Minimum inhibitory concentration (MIC) was defined as the lowest concentration of the ketone compounds at which no fungal growth was observed and lethal doses values (LD_25_) were calculated by interpolation in the above mentioned lineal regression. The *p* values and *R*^2^ parameters were used to check the validity of the regression analysis.

### 2.3. Molecular Modeling and Calculation of Molecular Parameters

The hydrophobic, electronic, polar, steric, geometric and topological descriptors were obtained from Herrera *et al.* [20].

### 2.4. Insect Growth

*S. zeamais* adults were obtained from Córdoba, Argentina. The colony was maintained in the laboratory for two year without exposure to insecticides. Insect rearing was carried under controlled temperature and humidity (28 °C and 60%–70%) and light/dark 12:12 h [21] on sterilized whole maize grain in sealed containers Adult weevils of mixed sex and age were used for the experiments. Experiments were conducted under complete darkness; temperature (28 °C) and relative humidity (60%–70%) remained constant in brood chamber.

### 2.5. Repellency Bioassays

Behavioral responses of *S. zeamais* adults to individual ketones were evaluated using a two-choice olfactometer, as described by Herrera *et al.* [17]. Briefly, a glass tube (30 cm × 1 cm of diameter) was connected to two Erlenmeyers (250 mL). In the center (15 cm from the ends) of a glass tube a small hole was made (1 cm × 1 cm). The connections were sealed with rubber plugs and they were covered with film to prevent gas leakage. The compounds were put on a filter paper (2 cm diameter) within each flask. Twenty insects deprived of food (24 h) were introduced into olfactometer (through the hole of the glass tube). After 2 h, we recorded the location of the insects in the flaks. Insects were given a choice between a specific dose of the test compound and the solvent (*n*-hexane) used as a control. The experiments being carried out between 10:00 and 16:00 h. Five replicates were performed for each assay. The position of the flasks was interchanged between consecutive tests to avoid spatial asymmetries. Insects was used once and afterwards discarded. Propionic acid was used as positive control [22].

A response index (RI) was calculated by using the equation:

RI = [(*T* − *C*)/Tot] × 100

Whereas *T* is number of insects responding to the treatment. *C* is number of insects responding to the control and Tot is the total number of insects released [23].

Negative values of RI indicate repellence to the treatment while positive ones indicate attraction.

### 2.6. Statistical Analysis

In the antifungal assay, the data were analyzed by one-way analysis of variance (ANOVA). The normality of the data was tested using the Shapiro-Wilk test. Comparisons between the control and treatment data sets were carried out using by the Di Rienzo, Guzmán and Casanoves Test (DGC test) [24] with results giving *p* values < 0.05 being considered significant.

Multiple linear regression analyses (MLR) were calculated in order to examine the quantitative relationships between linear combinations of the dependent variable (log 1/LD_25_) and the predictor variables (structure and molecular properties). Molar concentrations of the LD_25_ values were used for the MLR analyses. In the MLR equations, *N* is the number of data points, *r* is the correlation coefficient between observed values of the dependent variable and the values calculated from the equation, and *R*^2^ is the square of the correlation coefficient and represents the goodness of fit. The quantitative structure-activity relationship (QSAR) model was validated with the root mean square prediction error (RMSPE) obtained by the cross validation leave-one out procedure. Results with *p* values < 0.05 were considered significant.

The significance of the mean RI in each treatment of Repellency bioassays was evaluated by the Student’s *t*-test for paired comparisons [25]. The most significant positive or negative mean values of RI were first analyzed by an analysis of variance and subsequently ranked by using the Duncan multiple range test (*p* ≤ 0.05).

All analyses were performed using the InfoStat software Professional 2010 p [26].

## 3. Results

### 3.1. Antifungal Activity of Ketones

The antifungal effect of ketone compounds against *F. verticillioides* M 3125 was evaluated in a range of concentration between 0 and 3 mM. Thymoquinone was the most active compound followed by *S*-carvone and *R*-carvone (MIC values: 0.87 mM; 3.84 mM and 4.56 mM, respectively) and the inhibition was widely dependent upon the compound concentration. On the other hand, dihydrocarvone, camphor, menthone and α-thujone had no significant effect on *F. verticillioides* growth at the evaluated concentrations (Table 1).

**Figure 2:**
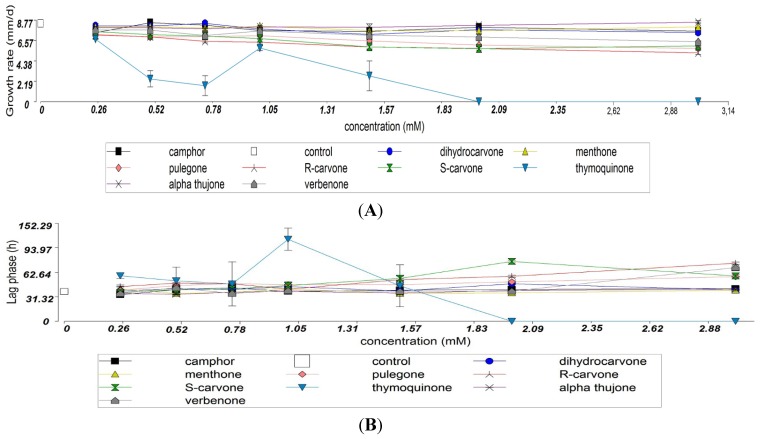
Effects of ketone compounds on the growth rate (**A**) and lag phase (**B**) of *F. verticillioides* M3125 strain.

The effect of ketones on growth rate is shown in Figure 2A. The results revealed that this growth parameter was affected depending on the compounds and the concentration in the culture medium. The most active ketone was thymoquinone showing a total inhibition of growth at concentration higher than 2 mM (*p* < 0.05). Moreover, the lag phase increased significantly with the amount of thymoquinone, pulegone and *R*-carvone (Figure 2B).

**Table 1:** Antifungal activity of ketone compounds against *Fusarium verticillioides* M3125 strain.

Compounds	MIC ^A^	LD_25_^B^	Inhibition (%) ± SD ^C^						
	Concentration (mM)								
			0.25	0.5	0.75	1	1.5	2	3
thymoquinone	0.87	0.23	23.7 ± 18.1 *^,d^	60.7 ± 41.1 * ^c^	89.7 ± 11.9 *^,a^	75.2 ± 18.6 *^,a^	85.3 ± 18.7 *^,a^	100 ± 0 *^,a^	99.4 ± 1.2 *^,a^
*R*-carvone	4.56	1.02	7.6 ± 10.0 ^e^	13.7 ± 9.3 ^e^	21.0 ± 5.5 *^,d^	22.3 ± 13.2 *^,d^	38.4 ± 7.6 *^,c^	45.3 ± 7.5 *^,c^	66.2 ± 8.3 *^,d^
*S*-carvone	3.84	1.48	0 ^f^	1.5 ± 2.9 ^f^	7.3 ± 8.9 ^f^	13.1 ± 11.3 ^e^	38.6 ± 12.1 *^,c^	58.8 ± 7.4 *^,b^	42.0 ± 11.1 *^,c^
pulegone	6.38	1.57	1.2 ± 1.4 ^f^	13.8 ± 5.3 * ^e^	10.9 ± 11.4 ^e^	15.7 ± 7.0 *^,e^	21.6 ± 10.0 *^,d^	34.5 ± 4.5 *^,c^	46.6 ± 5.8 *^,c^
dihydrocarvone	13.57	4.19	0 ^f^	0 ^f^	0 ^f^	0.6 ± 1.2 ^f^	1.2 ± 1.4 ^f^	8.6 ± 4.6 ^e^	2.3 ± 2.7 ^f^
menthone	ND	ND	0 ^f^	0 ^f^	0 ^f^	0 ^f^	1.5 ± 2.9 ^f^	0 ^f^	0 ^f^
verbenone	4.90	2.43	0 ^f^	1.2 ± 2.3 ^f^	1.8 ± 2.2 ^f^	0.6 ± 1.2 ^f^	1.5 ± 1.8 ^f^	4.9 ± 6.1 ^f^	44.6 ± 11.7 *^,c^
α-thujone	ND	ND	0 ^f^	0 ^f^	0.6 ± 1.2 ^f^	0 ^f^	0 ^f^	0 ^f^	0 ^f^
camphor	26.64	7.94	0 ^f^	0 ^f^	0 ^f^	0 ^f^	0 ^f^	0 ^f^	4.3 ± 5.0 ^f^

^A^ Minimal inhibitory concentration (MIC); ^B^ The lethal doses_25_ (LD_25_). The LD_25_ values were used in multiple linear regression analyses (MLR); ND: No determinate; ^C^ Inhibition of fungal growth was determined after 7 days of incubation. Values are expressed as means ± SD; * Indicate significant difference with the control and values having different letters are significantly different from each treatment according to DGC test of multiple range (*p* < 0.05). The experiments were performed twice in triplicate.

### 3.2. Molecular Modeling and Calculation of Molecular Parameters

The analysis was carried out using LD_25_ of thymoquinone, carvone, pulegone, dihydrocarvone, verbenone and camphor, in order to determine which physicochemical and topological characteristics (Table 2 and Table 3) were better at inhibiting *F. verticillioides* growth. The LD_25_ values were calculated by interpolation in the above-mentioned lineal regression. The LD_25_ values of dihydrocarvone and camphor were outside of the concentration evaluated range.

Equation (1) expresses these results:

Log (1/LD_25_) = 0.17 (Pi Energy) + 0.64 (1)

*R*^2^ = 0.95, RMSPE = 13.9%, *p* = 0.001, *N* = 6.

**Figure 3:**
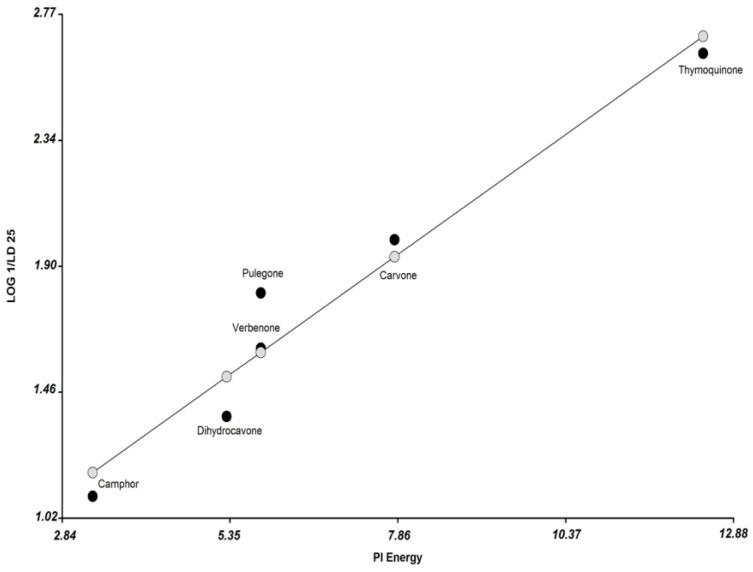
Plot of observed values (1/LD_25_) *vs.* the descriptor Pi Energy. The observed values of the ketones compounds are shown in black circle, and the predicted values (according to Equation (1)) are shown in grey circle.

In Equation (1) the relation between observed and predicted activity of the ketone compounds is shown in Figure 3. The obtained model showed a prediction error of 13.9% (RMSPE = 13.9%). Thus, an increase in the value of Pi energy represents a greater antifungal activity.

A second QSAR analysis was performed in order to include the ketones menthone and α-thujone, which did not show antifungal activity at the tested concentrations (>3 mM). Subsequently, we arbitrarily assumed that the LD_25_ of the inactive compounds was 11.89 mM (outside the concentration range tested), and these compounds were included in the new MLR analysis, Equation (2):

Log (1/LD_25_) = 0.19 (Pi Energy) + 0.45 (2)

*R*^2^ = 0.94, RMSPE = 18.3%, *p* = 0.0001, *N* = 8.

**Table 2:** Physicochemical descriptors used in the QSAR study.

Compounds	Dreiding Energy kcal/mol	Dipolo (Debye)	Molar Refractivity	Surface Tension (dyne/cm)	Volume (A^3^)	Minimal Projection Area (A^2^)	Maximal Projection Area (A^2^)	Molar Volume (cm^3^)	Log P	Pi Energy	Boiling Point C HectoPascal	Polarizability ×10^−24^ cm^3^)	Enthalpy of Vaporization (Kj/mol)	Polar Surface Area (A^2^)	Solvent Accessible Surface Area	pKa
thymoquinone	34.46	0.12	48.89	35.8	159	32.29	53.09	154.1	2.33	12.42	309	18.1	46.9	34.14	245.95	−7.75
carvone	35	3.2	47.17	29.8	156.9	31.02	50.97	159.8	2.27	7.81	307	18	46.7	17.07	248.75	−4.66
pulegone	40.89	2.88	47.13	29.5	164.5	31.96	53.06	164.8	2.56	5.81	298	18.2	46	17.07	277.53	−4.43
dihydrocarvone	27.07	2.79	46.3	27.98	164.9	31.45	51.84	168.47	3.22	5.3	294	18.19	45.79	17.07	274.86	−7.42
menthone	27.39	2.73	46.52	27.3	172.6	30.28	57.81	175.1	2.63	3.3	273	18.4	44.1	17.07	300.97	−7.42
verbenone	92.48	3.79	45.37	29.46	154.3	34.84	46.84	151.4	2.14	5.81	302	17.48	46.41	17.07	253.13	−4.73
thujone	146.8	2.76	44.54	35	160.1	34.23	47.95	150.8	1.9	3.3	266	17.6	43.7	17.07	283.79	−7.42
camphor	51.95	3.02	44.49	31.6	161.3	37.48	42.7	154.9	2.13	3.3	275	17.6	44.4	17.07	284.25	−7.49

The descriptors were obtained by [20].

**Table 3:** Topological descriptors used in the QSAR study.

Compounds	Platt Index	Randic Index	Balaban Index	Harary Index	Wiener Index	Hyper Wiener Index	Wiener Polarity	Szeged Index
thymoquinone	34	5.52	2.55	30.7	187	417	17	292
Carvone *	30	5.11	2.18	26.5	152	337	17	240
pulegone	30	5.11	2.22	26.6	150	327	14	236
dihydrocarvone	30	5.11	2.18	26.5	152	337	14	240
menthone	30	5.11	2.22	26.6	150	327	14	236
verbenone	40	5.03	2.2	28.58	132	252	16	272
thujone	40	5.08	2.13	28.05	139	283	15	169
camphor	42	4.98	2.4	29.58	123	219	19	174

The descriptors were obtained by [20]; * the chirality was not taken into account.

**Figure 4:**
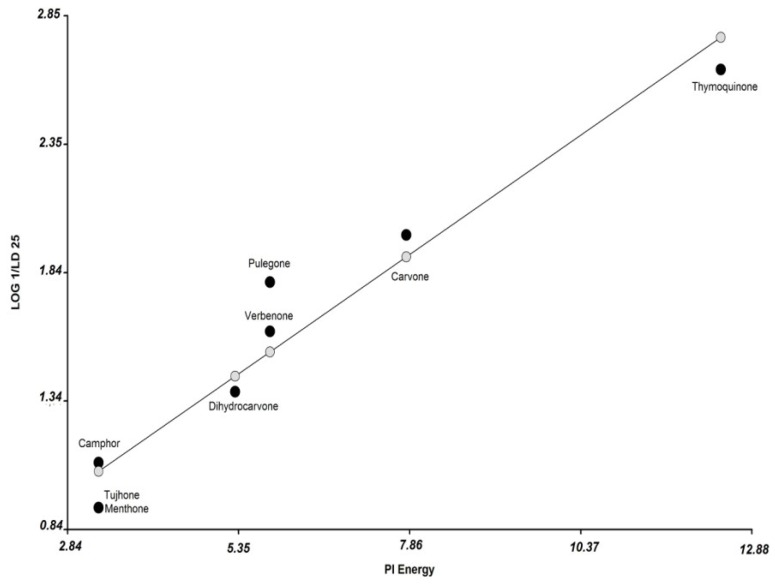
Plot of observed values (1/LD_25_) *vs.* the descriptor Pi Energy. The observed values of the ketones compounds are shown in black circle, and the predicted values (according to Equation (2)) are shown in grey circle.

The Equation (2) is shown in Figure 4. The obtained model showed a value of prediction error (RMSPE = 18.3%) higher than in Equation (1). Also, an increase in the value of Pi energy representing a greater antifungal activity is shown above.

### 3.3. Repellency Bioassays

**Figure 5:**
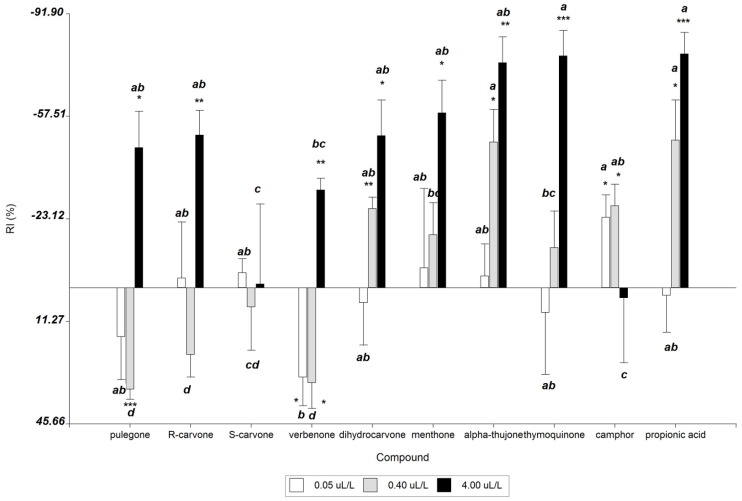
Behavioural responses of *S. zeamais* adults to ketone terpenes. * *p* ≤ 0.05; ** *p* < 0.01; *** *p* < 0.001 (significant response to experimental stimulus; paired-sample *T* test). Values having different letters are significantly different from each treatment according to Duncan’s multiple range test at *p* ≤ 0.05 (*n* = 5); (−) values of RI indicate repellence; (+) values of RI indicate attraction.

The behavioural response of *zeamais* weevils to ketones is presented in Figure 5. Except for *S*-carvone and camphor, all tested ketones showed repellent effects on *S.* *zeamais* at the dose of 4 µL/L. Thymoquinone and α-thujone were strong repellents (−77.8 ± 8.5. *p* < 0.001; −75.5 ± 8.6. *p* = 0.001, respectively), with similar values to the positive control propionic acid. At 0.4 µL/L, pulegone and verbenone showed significant attraction effects (34.1 ± 3.3. *p* < 0.001; 31.9 ± 8.6. *p* = 0.02, respectively) while dihydrocarvone, α-thujone and camphor were significant repellents. In contrast, at 0.05 µL/L verbenone showed significant attraction effects (30.1 ± 9.5. *p* = 0.03).

On the other hand, dihydrocarvone, menthone, α-thujone and thymoquinone showed direct relation between concentration and repellent insect (*p* < 0.05), but they did not show significant repellent effects at low concentration.

## 4. Discussion

Considering the impact of synthetic pesticides on human health and ecosystems, the use of eco-friendly compounds becomes necessary to protect stored-products. In this study, antifungal and repellent activities of nine ketones were evaluated against two stored-pests. Few studies report the antifungal activity of pure ketones and essential oils rich in these compounds against *F. verticillioides* [27,28]. Thymoquinone was the most active ketone against *F. verticillioides* having significant effect on growth parameters. Similar results were obtained by phenolic compounds [14,15,29,30].

The model obtained in Equations (1) and (2) reveal that a rise in Pi energy values increase the antifungal activity of ketone compounds against *F. verticillioides* M3125. This electronic descriptor was calculated by the Huckel method and is related to the localization energies for electrophilic and nucleophilic attraction in a molecule [31]. The number of double bonds present in ketones increases the values of Pi energy.

In this study, α,β-unsaturated ketones (thymoquinone, *R*–*S* carvone, pulegone and verbenone) were the most inhibitory compounds. These chemicals presented an extra double bond to the carbonyl group, therefore the Pi energy values are greater than saturated ketones. The α,β-unsaturated ketones could present keto-enol tautomerism, referring to the interconversion of the two forms involves the movement of a α-hydrogen and the shifting of bonding electrons [32,33,34,35,36]. So, these compounds might react by mechanism such as Michael reaction, *etc*. On the other hand, the toxicity of α,β-unsaturated ketones is dependent on the substituents at the β-carbon [31,32,33,34]. Verbenone and pulegone present alkyl substituents at β-carbon. Hence, this could explain their lowest antifungal effect than thymoquinone and *R*–*S* carvone [33].

Furthermore, the behavioral responses of *S. zeamais* adults to individual ketones evaluated using a two-choice olfactometer revealed that thymoquinone and thujone were the best repellents at higher concentrations, while camphor was repellent at low concentrations. In contrast, pulegone and verbenone acted as attractants at low concentrations but repellents at higher concentrations. In previous studies, some volatile compounds from fungi and grain showed attractant effects at low concentrations and repellent effects at higher concentrations against *S. granarius* and *S. zeamais*, thus, showing the ability of the species to detect and respond differentially to variations in concentration [17]. In addition, previous studies reported from our group showed high insecticidal activity of terpene ketones against *S. zeamais* [16,35].

Here, the antifungal effect of eight natural ketone compounds on *F. verticillioides*, and the repellent effect against their insect vector, *S. zeamais* was reported. However, the antifungal effect of the natural compounds is not necessarily related tothe anti-fumonisin effect. The mycotoxins are secondary metabolites, and their production is dependent on the stress condition. So, when the antifungal compounds are applied at sub-lethal concentrations, the fungus could reach a stress state, and mycotoxin production could be stimulated. The stimulation of fumonisin production by natural products has been previously reported [37]. Further studies will be carried out in order to determine and predict the effect of the evaluated compounds on fumonisin production.

## 5. Conclusions

The results obtained in the present work showed the capacity of thymoquinone as a potential tool for controlling *F. verticillioides* M3125, due to its antifungal activity, and repellent effect against the insect fungal vector, *S. zeamais*. In addition, the QSAR model revealed that the antifungal activity of ketone compounds could be predicted by the electronic descriptor, Pi energy, revealing the importance of α,β-unsaturation in the antifungal activity of these ketones. This study, contributes to the search of novel active compounds to control two storage pests of maize.
